# The Association of Virulence Determinants of Uropathogenic *Escherichia coli* With Antibiotic Resistance

**DOI:** 10.5812/jjm.9936

**Published:** 2014-05-01

**Authors:** Sara Asadi, Mohammad Kargar, Kavous Solhjoo, Akram Najafi, Sadegh Ghorbani-Dalini

**Affiliations:** 1 Department of Parasitology, School of Medicine, Jahrom University of Medical Sciences, Jahrom, IR Iran; 2 Department of Microbiology, Jahrom Branch, Islamic Azad University, Jahrom, IR Iran; 3 Department of Marine Microbiology, The Persian Gulf Marine Biotechnology Research Center, Bushehr University of Medical Sciences, Bushehr, IR Iran; 4 Department of Microbiology, Jahrom Branch, Young Researcher's Club, Islamic Azad University, Jahrom, IR Iran

**Keywords:** *Escherichia coli*, Virulence Factor, Drug Resistance

## Abstract

**Background:**

The emergence of antimicrobial resistant strains of *Escherichia coli* has raised considerable interest in understanding the diversity and epidemiology of *E. coli* infections in humans. Virulence factors of *E. coli* determine the specific infections caused by this microorganism.

**Objectives:**

This study aimed to determine the prevalence of eight *E. coli* virulence factors and their association with antimicrobial resistance in bacteria isolated from patients with urinary tract infections (UTI).

**Patients and Methods:**

One thousand patients with UTI were enrolled in this cross-sectional study. Antimicrobial susceptibility was examined by disc diffusion method according to CLSI guidelines. After DNA extraction, the materials were probed by PCR for eight virulence factors genes, namely *fimH*, *hly*, *iucC*, *ibeA*, *sfa*/*foc*, *neuC*, *papC,* and *afa* genes.

**Results:**

The frequency of virulence factors *papC*, *afa*, *sfa*/*foc*, *fimH*, *hly*, *neuC*, *ibeA,* and *iucC* were 53.3%, 51.7%, 53.3%, 56.7%, 23.3%, 31.7%, 20%, and 73.3%, respectively. In addition, there was a high degree resistance to cotrimoxazole and nalidixic acid while a high degree of susceptibility to nitrofurantoin was detected. There was a statistically significant association between *fimH* gene and resistance to ciprofloxacin (P = 0.006), nalidixic acid (P = 0.025), and cotrimoxazole (P = 0.02). Such associations were found between *ibeA* gene and amikacin (P = 0.02) and cotrimoxazole (P = 0.02) as well as between *afa* gene and gentamycin (P = 0.05).

**Conclusions:**

The results showed that *E. coli* isolated from patients with UTI had eight virulence factors with high frequencies. Moreover, these results alleged a direct connection between virulence factors and antimicrobial resistance in *E. coli*.

## 1. Background

The incidence of urinary tract infection (UTI) is estimated to be about 150-250 million cases worldwide. It also accounts for approximately 35% of all hospital acquired infections (1, 2). *Escherichia coli* is one of the most common agent causing extra intestinal infections. These infections are an important cause of morbidity, mortality, and increased healthcare costs. In addition, they are common leading causes of UTI, pneumonia, meningitis, osteomyelitis, sepsis, and intra-abdominal as well as diverse soft tissue infections. 

*E. coli* strains causing UTI are termed uropathogenic *E. coli* (UPEC). UPEC isolates are a genetically heterogeneous group that possess several virulence factors (VFs) necessary for persistence and colonization of the bacteria in the urinary tract, overcome host defenses, and extra intestinal disease (3-5). These VFs include fimbrial adhesins (P, type 1, S, and F1C fimbriae), afimbrial adhesin, toxins (hemolysin and cytotoxic necrotizing factor), siderophores (aerobactin system), and capsular polysaccharide (group II capsules) (5-7).

Non-complicated infections constitute the majority of UTIs. Patients recognized with acute non-complicated cystitis are treated as outpatients. The microbiological features of this infection are greatly predictable even in healthy subjects. Therefore, physicians have been informed that empirical antibiotic treatment without culture is convenient in such cases. The empirical therapy has been so widely used that only a few UTIs are routinely cultured (1). Worldwide data shows that there is an increasing resistance to conventional drugs among UTI pathogens. Resistance has emerged even to the newer and more potent antimicrobial agents. Antimicrobial resistance surveillance is necessary in order to determine the significance of the problem and to guide empirical selection of antimicrobial agents to treat infected patients (2).

During the last few decades, the frequency of antibiotic resistant infections have raised permanently around the world. This increase has been attributed to a combination of the selective pressure of antimicrobial use, microbial characteristics, social and technical changes that accelerate the transmission of resistance factors in microorganisms including misuse and increased use of antibiotics, a higher number of susceptible hosts, and mistakes in infection control programs leading to incremented transmission of resistant microorganisms (8).

However, several investigations have reported the opposite results. Their studies have shown that according to clinical feature, virulence factor, and antibiotic resistant profile, some *E. coli* strains tend to be less virulent than susceptible isolates. Furthermore, it remains unclear whether such trends are owing to causal relationships between virulence and resistance or result from co-associated factors (3).

## 2. Objectives

This study was conducted to assess the prevalence of VFs and their association with the antibiotics resistance in patients with UTI.

## 3. Patients and Methods

### 3.1. Sample Collection

In this cross-sectional study, 1000 urine samples from patients referred to Peimanieh Hospital Laboratory in Jahrom between 2010 and 2011 were examined. In addition, basic characteristics data such as age, sex, history of urinary infection, history of antibiotic usage, and history of any hospitalization during the past 28 days were recorded. Written informed consent was obtained from the patients or guardian of each child. Ethic Committee of Islamic Azad University approved all steps of this study.

Standard media, including blood agar and MacConkey agar (Merck, Germany) were used for pathogen isolation. Identification of all isolates was done on the basis of Gram staining and routine biochemical tests including fermentation of lactose, citrate utilization, motility of organism, ability to produce indole, reaction on triple sugar iron (TSI) medium, and hemolysis on blood agar. The organisms were stored at 4 °C on agar slants and at -20 °C in glycerol for further investigations. DNA was extracted from confirmed *E. coli* strains by using a DNA extraction kit (DNP^TM^ kit, CinaGen Co., Iran) according to the manufacturer's instructions. The quality of extracted DNA was examined by 1% agarose gel electrophoresis and concentration of extracted DNA was analyzed by A260/A280 ratio in a biophotometer (Eppendorf, Germany).

### 3.2. PCR Method to Determine Virulence Factors

**Figure 1.**
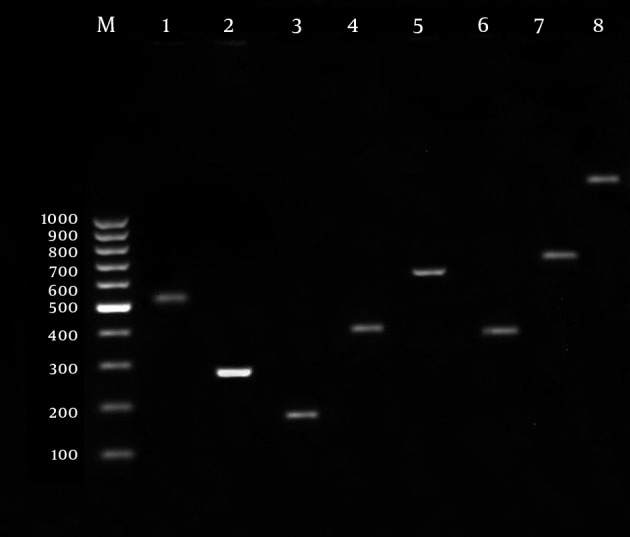
Gel Electrophoresis of Virulence Genes of Uropathogenic *E. coli*. M) Size Marker (100 bp); 1) fimH (508 bp); 2) iucC (269 bp); 3) ibeA (171 bp); 4) sfa/foc (410 bp); 5) neuC (675 bp); 6) papC (328 bp); 7) afa (750 bp); 8) hly (1117 bp);

For identification of VFs, 2 µL of extracted DNA was amplified with 1.5 mM MgCl_2_, 0.2 mM deoxynucleoside triphosphates (dNTPs) mixture, 0.2 mM of each primer (Table 1), and 1 U of *Taq* DNA polymerase (CinaGen, Co., Tehran, Iran). The PCR was performed with a Perkin-Elmer Gene Amp 9600 thermal cycler under the following conditions: initial denaturation for 5 min at 94 °C followed by 30 cycles of 30 s at 94 °C, 30 s annealing at the specific melting temperature of each primer (Table 1), 30 s at 72 °C, and a final extension step of 7 min at 72 °C. The amplified products were visualized after electrophoresis on a 1.5% agarose gel stained with ethidium bromide (Figure 1).

**Table 1.** The Sequence of Primers and Size of Amplified Products (6)

Gene Sequences	Size, bp	Tm, °C
***fimH***	508	55
F: TGCAGAACGGATCCGTGG		
R: GCAGTCACCTGCCCTCCGGTA		
***iucC***	269	55
F: AAACCTGGCTTACGCAACTGT		
R: ACCCGTCTGCAAATCATGGAT		
***papC***	328	65
F: GACGGCACTGCTGCAGGGTGTGGCG		
R: ATATCCTTTCTGCAGGGATGCAATA		
***sfa/foc***	410	65
F: CGGAGGAGTAATTACAAACCTGGCA		
R: GAGAACTGCCCGGGTGCATACTCT		
***ibeA***	171	60
F: TTACCGCCGTTGATGTTATCA		
R: CATTAGCTCTCGGTTCACGCT		
***neuC***	675	61
F: AGGTGAAAAGCCTGGTAGTGTG		
R: GGTGGTACATTCCGGAGTGTC		
***hly***	1177	63
F: AACAAGGATAAGCACTGTTCTGGCT		
R: ACCATATAAGCGGTCATTCCCGTCA		
***afa***	750	65
F: GCTGGGCATCAAACTGATAACTCTC		
R: CATCAAGCTGTTTGTTCGTCCGCCG		

### 3.3. Antimicrobial Susceptibility Testing

The antimicrobial susceptibilities pattern of *E. coli* strains were assessed using the disk diffusion method on Mueller–Hinton agar (Biorad, Marnes-la-Coquette, France) according to the Clinical and Laboratory Standards Institute (9).

In the present study, the eight used antibiotics were as follows: cotrimoxazole (1.25 μg trimethoprim and 23.75 μg sulfamethoxazole), nalidixic acid (30 μg), ciprofloxacin (5 μg), cefixime (5 μg), gentamicin (10 μg), cephalexin (30 μg), amikacin (30 μg), and nitrofurantoin (300 μg) (Mast Diagnostics, Merseyside, UK). *E. coli* strain ATCC 25922 was used as quality control.

### 3.4. Statistical Analysis

Data were statistically analyzed using SPSS v.15 (SPSS Inc., Chicago, IL, USA). Descriptive analyses were done to the evaluation of parametric and non-parametric variables. In addition, caramer, fi, and logistic regression were performed to assess of variables correlation. P value < 0.05 was considered as statistically significant.

## 4. Results

In total, 116 out of 1000 samples showed bacterial growth in culture media including 60 (51.7%) *E. coli*, 43 (37.1%) *Klebsiella*, 8 (6.9%) *Pseudomonas*, and 5 (4.3%) *Proteus*. Among 60 UPEC isolates, 47 isolates were from females and 13 were from males. The mean age of the children studied was 33.8 ± 2.07 years old; the oldest patient had 90 years of age and the youngest one was one year old. The frequency of each virulence factor was as follows: 19 (31.7%) *neuC* (K1capsular antigen), 31 (51.7%) *afa* (afimbriae adhesion), 32 (53.3%) *sfa*/*foc* (type S fimbriae), 34 (56.7%) *fimH* (type I fimbriae), 32 (53.3%) *papC* (type P pilli), 14 (23.3%) *hly* (alpha-Hemolysin), 12 (20%) *ibeA* (invasive protein A), and 44 (73.3%) *iucC* (aerobactin system). 

The rate of resistance to cotrimoxazole, nalidixic acid, ciprofloxacin, cefixime, gentamycin, cephalexin, amikacin, and nitrofurantoin antibiotics were 45%, 41.7%, 21.7%, 20%, 11.7%, 16.7%, 13.3%, and 3.3%, respectively (Table 2). Statistical analysis revealed the existence of the following associations: between *fimH* gene and resistance to ciprofloxacin (P = 0.006), nalidixic acid (P = 0.025), and cotrimoxazole (P = 0.02); between *ibeA* gene and amikacin (P = 0.02) and cotrimoxazole (P = 0.02); *afa* gene and gentamycin (P = 0.05) (Table 3). Moreover, there was an association between the *hly* gene and a history of antibiotic consumption (P = 0.04).

**Table 2.** Antibiotic Susceptibility Pattern of Uropathogenic *E. coli *^a^

Antibiotics	Sensitive	Resistant	Intermediate
**Ciprofloxacin**	44 (73.3)	13 (21.7)	3 (5)
**Nalidixic acid**	34 (56.7)	25 (41.7)	1 (1.7)
**Amikacin**	48 (80)	8 (13.3)	4 (6.7)
**Cotrimoxazole**	31 (51.7)	27 (45)	2 (3.3)
**Nitrofurantoin**	58 (96.7)	2 (3.3)	0
**Cefixime**	46 (76.7)	12 (20)	2 (3.3)
**Gentamycin**	48 (80)	11 (11.7)	5 (8.3)
**Cephalexin**	45 (75)	10 (16.7)	5 (8.3)

^a^ Data are presented in No. (%).

**Table 3.** Distribution of Antimicrobial Resistance Among Virulence Factors ^a^, ^b^

Antibiotic	CN	GM	CFM	FM	SXT	AN	NA	CP
**neuC** **(n = 19)**								
Positive, %	2 (10.5)	2 (10.5)	2 (10.5)	0 (0)	9 (47.4)	2 (10.5)	9 (47.4)	2 (10.5)
P value	0.38	0.85	0.91	0.32	0.8	0.66	0.54	0.15
***Afa*** ** (n = 31)**								
Positive, %	5 (16.1)	6 (19.4)^ c^	8 (25.8)	2 (6.5)	14 (45.2)	4 (12.9)	13 (41.9)	6 (19.4)
P value	0.91	0.05	0.24	0.16	0.97	0.91	0.96	0.65
***Sfa/foc*** ** (n = 32)**								
Positive, %	6 (18.8)	4 (12.5)	6 (18.8)	0	13 (40.6)	2 (6.3)	13 (40.6)	6 (18.8)
P value	0.64	0.83	0.79	0.12	0.46	0.08	0.86	0.55
***fimH*** ** (n = 34)**								
Positive, %	4 (11.8)	4 (11.8)	4 (11.8)	1 (2.9)	11 (32.4)^c^	6 (17.6)	12 (35.3)^c^	3 (8.8)^c^
P value	0.24	0.97	0.06	0.84	0.02	0.26	0.025	0.006
***papC*** ** (n = 32)**								
Positive, %	6 (18.8)	3 (9.4)	8 (25)	1 (3.1)	16 (50)	3 (9.4)	14 (43.8)	8 (25)
P value	0.64	0.55	0.3	0.92	0.4	0.33	0.72	0.5
***hly*** ** (n = 14)**								
Positive, %	1 (7.1)	2 (14.3)	2 (14.3)	0	5 (6.3)	2 (14.3)	3 (21.4)	1 (7.1)
P value	0.27	0.72	0.54	0.42	0.42	0.9	0.07	0.13
***ibeA*** ** (n = 12)**								
Positive, %	3 (25)	2 (16.7)	3 (25)	0	9 (0.75)^c^	4 (33)^c^	6 (50)	3 (25)
P value	0.38	0.54	0.62	0.47	0.02	0.02	0.51	0.75
***iucC*** ** (n = 44)**								
Positive, %	8 (18.2)	6 (13.6)	10 (22.7)	1 (2.3)	19 (43.2)	5 (11.4)	20 (45.5)	10 (22.7)
P value	0.6	0.43	0.38	0.44	0.63	0.45	0.32	0.74

^a^ Abbreviations: AN, Amikacin; CFM, Cefixime; CN, Cephalexin; CP, Ciprofloxacin; FM, Nitrofurantoin; GM, Gentamycin; NA, Nalidixic acid; SXT: Sulfamethoxazole with Trimethoprim (cotrimoxazole).

^b^ Data are presented in No. (%).

^c^ Statistically significant

## 5. Discussion

*E. coli* is the leading causative agent of UTI and one of the most important bacterial infections. In most cases, uropathogenic clones originate from the fecal flora, and the pathogenic potential of *E. coli* isolates is thought to be dependent on the presence of various VFs (5). This study showed that there was a difference in rate of VFs in 60 examined UPEC.

Tiba et al. (5) conducted a study on the genetics of VFs of pathogenic *E. coli* from patients with cystitis. The highest frequency rates of VFs were consecutively attributed to *fimH* (97.5%), *papC* (32.7%), *afa* (27.8%), *iucC* (25.9%), *hly* (25.3%), and *afa* (6.2%). Moreover, the incidence of virulence genes in *E. coli* strains isolated from Romanian adult with UTI was *fimH* (86%), *sfa*/*foc* (23%), *papC* (36%), and *afa* (14%) (7). Andreu et al. (10) identified the presence of fimbriae type I (*fimH*) in *E. coli* strains isolated from patients with pyelonephritis, cystitis, and recurrent UTI as 97%, 97%, and 90%, respectively. In addition, the frequency rate of *papC* was 73%, 0%, and 20%, respectively. In another study, Yasuoka reported that the most frequent *E. coli* VFs in stool, urine, and blood samples in Japan were *ibeA *(44%), *papC* (45%), and *hly*A (22%) (11). 

In a further study by Santo, which was performed on VFs of pathogenic *E. coli* strains, the prevalence of VFs were reported as *sfa* 19%, aerobactin 76%, *papC* 11%, *afa* 32%, and hemolysin 96% (12).Blanco reported that the frequency rates of *sfa*, *papC,* and *afa* VFs in pathogenic strains of *E. coli* isolated from patients with UTI were 53%, 54%, and 2%, respectively (13).

In another study applied by Farshad et al. (14), the prevalence rates of *sfa*, *papC,* and *hly* VFs in children with UTI were 13.5%, 22.9%, and 14.6%, respectively. Benton examined *E. coli* VFs of patients with spinal injuries. The prevalence rates of VFs were as follows: antigen K (28%), aerobactin (39%), fimbraie P (17%), and hemolysin (27%) (15).

The differences observed in the prevalence of *E. coli* VFs in this study and reports from some regions (7, 11, 12, 14) could be explained by the sample type, source, size, type of VFs, and geographic location. Horcajada showed that nalidixic acid resistance was significantly associated with a decrease in prevalence of three VFs, namely *sfa*, *hly,* and *cnf-1* (16). In another study, Arisoy et al. (17) showed an association between susceptibility to antibiotics and VFs of the *E. coli* isolates causing pyelonephritis. They reported that virulence genes pap, *sfa*, *afa*I, *hly,* and *aer* were increased in sensitive strains. 

Zhao examined the prevalence of VFs and antibiotic resistance in UPEC. The *feoB* and *fimH* genes had the highest prevalence. in addition, among the 15 tested antibiotics for resistance, nalidixic acid, mezlocillin, and tetracycline had the most resistance (18). In this study, stronger associations were found between *fimH* gene and resistance to ciprofloxacin, nalidixic acid, and cotrimoxazole, between *ibeA* gene and amikacin and cotrimoxazole, and between *afa* gene and gentamycin. These findings were similar to those reported in other studies (16-18).

Antimicrobial susceptibility patterns varied in isolates from different categories of patients. it needs to be considered when developing guidelines for treatment of UTI and interpreting data from other published studies, which showed high prevalence of antimicrobial resistance among UPEC. From our data, older drugs like nitrofurantoin appeared to be useful and could be considered as a choice for treating uncomplicated lower UTI.

To sum up, *E. coli* causing UTI in different patient populations differ in their pathogenic potential and susceptibility to antimicrobial agents. It has to be considered when developing guidelines for management of UTI. Periodic reviews and formulation of antibiotic consumption policy are required to control the transmission and acquisition of antibiotic resistance. Further studies for better understanding of the interaction between different VFs at a molecular level are necessary as most UPEC isolates simultaneously express several VFs.
